# Factors associated with good health among older persons who received a preventive home visit: a cross-sectional study

**DOI:** 10.1186/s12889-020-08775-6

**Published:** 2020-05-14

**Authors:** Anna Nivestam, Albert Westergren, Pia Petersson, Maria Haak

**Affiliations:** 1 Department of Nursing and Health Sciences, Kristianstad University, SE-291 88 Kristianstad, Sweden; 2 Department of Health Sciences, Lund University, Box 157, 221 00 Lund, Sweden

**Keywords:** Self-rated health, Health promotion, Physical, Mental, Lifestyle, Older adult, District nurse, Society, Community dwelling older people, Healthy ageing

## Abstract

**Background:**

The ageing population is increasing worldwide, and this trend is bringing challenges both for the older person and for society. In order to meet the challenges a comprehensive approach is needed involving both health promotion and risk prevention. The preventive home visit is a public health intervention used around the world with the purpose of promoting health and preventing risk among older persons. However, most preventive home visits are focused on questions asking about risks. In order to strengthen the health promotion perspective during the preventive home visits, factors associated with good health need to be identified. The aim of this study was therefore to determine which factors were associated with good self-rated health among older persons who received preventive home visit.

**Methods:**

This was a register study with a cross-sectional design, including older persons (≥75 years old), living in their own homes, and that had received preventive home visit. Data were collected during a period of 9 months, in two municipalities in the south of Sweden. A questionnaire covered mental, physical and lifestyle factors were used at home visit. Binary logistic regression was used to analyse the data.

**Results:**

In total, 619 older persons were included in the study; 55.4% were women, and the mean age was 80.6 years (standard deviation 2.2 years). The following items were significantly associated with good health (after adjustment for age and gender): being able to do things that make one feel valuable, having no physical problems affecting participation in social activities, not feeling sad, not having reduced energy, and not having impaired endurance.

**Conclusions:**

The main conclusion of this study is that questions focusing on risks could be seen from a health promotion perspective and could thus be turned into assets with a positive impact on older persons’ health. Furthermore, the mental and physical factors identified in the results as associated with good health have implications for the person’s ability to feel valuable and participate in social activities. The results suggest that issues regarding both health promotion as well as risk prevention must be brought up during the preventive home visit.

## Background

The ageing population is increasing worldwide [1], and this trend is bringing challenges both for the older person and society. With an ageing population, society can expect increased demands on health services [2] and older persons can face mental [3], social [4] and physical challenges [5]. In order to meet the challenges, older persons as well as society need to consider how to promote healthy ageing. According to Bauer et al. [6] there is a need for a comprehensive approach considering both health promotion and risk prevention to achieve good health in the population. One approach used to highlight positive aspects of health and to identify factors which contribute to good health in the population is the asset-based approach [7]. This approach focuses on factors that can contribute to good health on both individual and societal levels [7]. Health assets can be factors such as social networks, a sense of purpose, and accessible environments [8]. The asset-based approach aims to enable a comprehensive perspective to empower older persons and society to improve and maintain good health in old age [7, 9].

In 2015 the United Nation launched the sustainable development goals (SDG) for 2030, the emphasis is on health promotion to enable good health and wellbeing for all [10, 11]. Health is defined by Huber et al. as an ability to adapt and self-manage [12]. Three decades ago the World Health Organization (WHO) stated in the Ottawa Charter that health promotion should be in focus in order to help a person to stay healthy, identify needs, and have the power to make changes to improve health or cope with the life situation [13]. However, substantial research has focused on younger people, for example mental [14, 15] and oral health promotion to adolescents [16, 17]. Meanwhile, research on older persons has focused merely on risk prevention [18] such as falling [19], malnutrition [20] and ulcer prevention [21] to avoid illness. However, to enable healthy ageing there is a need to focus on health promotion as well as risk prevention.

The preventive home visit (PHV) to older persons is an example of a public health intervention that includes both health promotion and risk prevention, and it has the aim of helping older persons to maintain good health [22]. Previous studies have shown that PHVs can delay mortality [23–25], decrease functional decline [24] and reduce the cost of health care [26]. PHVs have been used worldwide [22] mainly to identify risks among older persons from different perspectives such as physical, mental and social [27]. However, since the aim of PHVs is comprehensive and also involves promoting health [22], more knowledge is needed about how a person’s assets and capabilities could be strengthened during the PHV even though the main focus of the questions is risk-based. Therefore, it is important to identify factors which could be turned into assets for older persons. In a review by Fagerstrom et al. [28] it was found that the PHV lacks a health promotion perspective and has a major focus on risk prevention. Recent research has focused on how risks could be prevented by the PHV [29]. In order to enable a comprehensive approach during the PHV and strengthen the health promotion perspective, there is a need to identify factors associated with good health. Thus, the aim of this study was to determine which factors were associated with good self-rated health (SRH) among older persons who received PHV.

## Methods

### Study design

A cross-sectional study design was used in this register study.

### Context

This study was conducted in two municipalities in northeast Skåne, Sweden that have long experience of offering PHVs to older persons living at home. Persons that are offered PHV have no or minimal home care and are aged 75 years or older. District nurses conduct the visits. Questions covering health issues are brought up in a dialogue with the older person and registered by the nurse. Geographically, the two municipalities cover both urban and rural areas. In 2018, the smaller municipality had about 660 persons in the age group 75–79 and about 470 persons aged 80–84. The corresponding figures for the larger municipality were 2460 and 1670 persons respectively [30].

### Data collection and sample

Anonymised data from the register was analysed in this study. Those included in the study were older persons who participated in the PHVs from January to September 2018 and answered a question about SRH, in total 619 persons (*n* = 280 and *n* = 339 persons from the larger and smaller municipalities respectively).

### Questionnaire

Questions selected from the register to be included in the present study were separated into those related to demographics, mental, physical and lifestyle factors (see Additional file 1). The questions were developed by health care professionals (including physicians and district nurses) and researchers, with the purpose of supporting the dialogue during the PHV. Some of the questions were inspired by validated risk assessment tools such as FRESH-screening [31], the Short Form Health Survey SF-36 [32], the Minimal Insomnia Symptom Scale MISS [33] and the Downton Fall Risk Index [34]. There were 36 items used in the present study (see Additional file 1). One of these questions focused on health and wellbeing, i.e. SRH. In research, SRH is a validated and well-used item for measuring health [35, 36] and a systematic review shows that good SRH could be a good indicator for health among older persons [9]. In the present study, wellbeing is integrated in the question about SRH, and this question does not separate the two (health and wellbeing). An assumption is made that this question rates general health.

### Analysis

Answers to the questions were dichotomised. Except the question “What year were you born?” which was presented as a continuous item, i.e. age. SRH with five response categories: Excellent; Very good; Good; Fair; and Bad was dichotomised into Good (the first three alternatives) and Poor (the last two alternatives). Independent bivariate analyses, i.e. comparing those with good and those with poor SRH, were performed with all the items described in the Additional file 1. Chi-square tests were used for all items, except those items where the sample size was under five in one of the cells of the 2 × 2 table, when Fisher’s exact test was used and a T-test was used for age to compare the two samples. The level of significance was set to *p*-values < 0.05. Binary logistic regression with SRH as the dependent item and all items with a p-value ≤0.2 from the bivariate analysis (Table 1) were used as independent items. Instead of an odds ratio that is commonly used in this kind of calculation, a positive odds ratio (POR) was used [37]. In a POR, the dependent and the independent items are positively coded. A POR > 1 was interpreted as: those rating their SRH as good are more likely than those with poor SRH to have a positive outcome of the independent item [37]. Logistic regression analysis was conducted in three steps. First a binary logistic regression forward model was analysed followed by an enter model. Age and gender were forced into the enter model together with the items that remained significant after the forward conditional analysis. This was done in order to adjust the PORs for age and gender. In the third and final model the non-significant items after adjustment for age and gender, “no digestive problems” and “feeling able to influence society” were excluded. Model fit was measured with the Hosmer and Lemeshow goodness-of-fit test, which measures differences between actual and predicted values of the dependent item. Good model fit is shown by a non-significant and small chi-square value, which together indicate no difference in actual and predicted dependent values [38]. Statistical analyses were conducted in the Statistical Package for the Social Science version 24.

**Table 1:** Characteristics of older persons (*n* = 619) in relation to good self-rated health as assessed during the preventive home visits

	Total ***n*** = 619	Self-Rated Health		***p***-value^**a**^
		Good *n* = 472	Poor *n* = 147	
**Demographics**				
Age, mean (SD)	80.6 (2.2)	80.5 (2.0)	80.7 (2.7)	0.395 ^b^
Female, n (%)	341 (55.4)	258 (54.8)	83 (57.2)	0.602
Settlement rural, n (%)	431 (71.6)	322 (70.0)	109 (76.8)	0.118
Cohabitant, n (%)	413 (67.4)	317 (67.7)	96 (66.2)	0.732
Satisfied with accommodation, n (%)	590 (97.2)	454 (98.3)	136 (93.8)	0.004
Feeling safe in the neighbourhood, n (%)	598 (98.7)	453 (98.3)	145 (100)	0.209^c^
Enough money to pay bills, n (%)	581 (96.8)	443 (97.1)	138 (95.8)	0.432
Not worried about own finances, n (%)	591 (98.0)	451 (98.3)	140 (97.2)	0.438
**Mental factors**				
Feeling serenity/safe/harmony, n (%)	514 (83.6)	424 (90.2)	90 (62.1)	< 0.001
Good cognition, n (%)	569 (93.7)	438 (94.8)	131 (90.3)	0.053
Good sleep, n (%)	527 (87.4)	414 (90.4)	113 (77.9)	< 0.001
Not feeling loneliness, n (%)	558 (91.2)	441 (94.2)	117 (81.3)	< 0.001
Not feeling sad, n (%)	478 (78.1)	404 (86.3)	74 (51.4)	< 0.001
Not feeling anxiety, n (%)	499 (83.2)	415 (90.6)	84 (59.2)	< 0.001
Not worried about the future, n (%)	490 (80.1)	403 (86.1)	87 (60.4)	< 0.001
Feeling influence over own situation, n (%)	593 (99.0)	453 (99.3)	140 (97.9)	0.151^c^
Feeling influence over society, n (%)	187 (31.4)	150 (33.1)	37 (25.9)	0.104
Satisfied with life, n (%)	502 (81.6)	423 (90.2)	79 (54.1)	< 0.001
Being able to do things that make one feel valuable, n (%)	514 (84.1)	435 (93.3)	79 (54.5)	< 0.001
Not having reduced energy, n (%)	536 (88.4)	440 (95.4)	96 (66.2)	< 0.001
**Physical factors**				
Not having impaired endurance, n (%)	531 (88.2)	437 (95.4)	94 (65.3)	< 0.001
Good ADL, n (%)	472 (78.1)	394 (85.5)	78 (54.5)	< 0.001
Having no physical problems affecting participation in social activities, n (%)	505 (82.8)	426 (91.6)	79 (54.5)	< 0.001
No pain, n (%)	364 (60.4)	306 (66.7)	58 (40.3)	< 0.001
Urinary continence, n (%)	538 (88.2)	423 (91.0)	115 (79.3)	< 0.001
No digestive problems, n (%)	535 (88.1)	429 (92.9)	106 (73.1)	< 0.001
Good vision, n (%)	449 (76.4)	347 (77.8)	102 (71.8)	0.145
Good hearing, n (%)	297 (64.1)	226 (66.3)	71 (58.2)	0.110
**Lifestyle factors**				
Physically active several times per week, n (%)	532 (89.9)	419 (93.3)	113 (79.0)	< 0.001
Good appetite, n (%)	567 (92.6)	448 (95.9)	119 (82.1)	< 0.001
No weight loss, n (%)	558 (91.2)	438 (93.8)	120 (82.8)	< 0.001
Rarely use alcohol, n (%)	299 (64.6)	209 (60.9)	90 (75.0)	0.006
Not smoking/using snuff, n (%)	482 (81.4)	359 (80.0)	123 (86.0)	0.105
Use a smartphone, n (%)	251 (41.9)	206 (45.4)	45 (31.0)	0.002
Use a computer, n (%)	342 (57.0)	272 (59.8)	70 (48.3)	0.015

*SD* Standard Deviation, *ADL* Activities of Daily Living, internal dropout varies between *n* = 3–156, ^a)^ Chi-Square test unless other stated; ^b)^ Independent sample T-test; ^c)^ Fisher’s exact test

### Ethical approval

The study was conducted in accordance with the Declaration of Helsinki [39] and approved by The Ethical Review Board, Lund, Sweden (reference number 2018/849).

## Results

The total sample included 619 older persons, with a mean (SD) age of 80.6 years (2.2 years). In the sample, 55.4% were women, 71.6% were living in rural areas, 67.4% were cohabitant, 97.2% were satisfied with accommodation, 98.7% felt safe in the neighbourhood and 96.8% felt that they had enough money to pay their bills. There were no significant differences between those with good SRH and the group with poor SRH concerning the demographic items except that those satisfied with their accommodation gave better SRH scores than those not satisfied with their accommodation (Table 1).

Older persons with good SRH reported significantly better mental factors. Compared to those with poor SRH they felt more serenity/safe/harmony, had good sleep, less loneliness, less sadness, less anxiety, fewer worries about the future, were more satisfied with their lives, were able to do things that made them feel valuable, and did not have reduced energy. Physical characteristics of the group with good SRH were not having impaired endurance, greater abilities in Activities of Daily Living, no physical problems affecting participation in social activities, no pain, being urinary continent, and no digestive problems. Furthermore those with good SRH reported lifestyle factors; they were physically active several times per week, had good appetite, no weight loss, rarely used alcohol, used smartphones and computers (Table 1).

The following items, sorted from highest to lowest POR, were significantly associated with good SRH after adjusting for age and gender: being able to do things that made one feel valuable, having no physical problems affecting participation in social activities, not feeling sad, not having reduced energy, and not having impaired endurance. The significant model explained almost 50% (Nagelkerke) of the variance in SRH (Table 2).

**Table 2:** Binary logistic regression (enter) model for items associated with good self-rated health adjusted for age and gender (*n* = 565)

	Good Self-Rated Health		
	POR	CI 95%	***p***-value
Age	1.02	0.91–1.14	0.794
Gender	0.85	0.51–1.43	0.542
Able to do things that make one feel valuable	5.72	3.01–10.87	< 0.001
No physical problems affecting participation in social activities	4.38	2.38–8.06	< 0.001
Not feeling sad	4.07	2.31–7.15	< 0.001
Not having reduced energy	3.44	1.37–8.65	0.009
Not having impaired endurance	2.91	1.18–7.19	0.021

*CI* Confidence Interval, *POR* Positive Odds Ratio. Hosmer-Lemeshow goodness-of-fit test, X^2^ = 10.50, *p* = 0.162; Nagelkerke R *p* = 0.462

## Discussion

The aim of the present study was to determine which factors were associated with a good SRH among older persons receiving PHV. It is important to identify factors associated with good health to enable a comprehensive approach, which might suggest activities that are oriented towards health promotion for older persons. The results showed that physical and mental factors, such as being able to do things that made one feel valuable, having no physical problems affecting participation in social activities, not feeling sad, not having reduced energy, and not having impaired endurance were associated with good health. The impact of mental and physical factors on health is well described in WHO’s World report on ageing and health [2]. However, factors such as no cognitive decline, no need for help with activities of daily living, and physical activity can also have an impact on healthy ageing [40]. Even though none of these factors were significant in the present study, they might still be important to consider during the PHVs in order to promote good health.

In the present study it seems that positive factors and factors that indicate the absence of problems could be associated with good health among older persons. For example, not feeling sad was positively associated with good health. It has been shown in a cross-sectional study from Brazil that poor health was associated with a low level of happiness and lack of energy among older persons [41]. However, the results from the present study do not indicate that a person necessarily has to feel happy in order to experience good health. Instead the results show that the absence of a problem per se can have a positive impact on health. Other factors shown in the present study that indicate the absence of a problem were for example not having reduced energy and no impaired endurance, which were also associated with the experience of good health. Although not asking specifically about feeling energetic one might conclude that having energy and endurance is good, based on literature that acknowledges lack of energy to be an indicator of poor health [5, 42]. In addition, a metasynthesis which explored older persons’ perception of health described having energy as an important factor [43]. However, the present results do not mean that older persons have to feel energetic or have increased endurance per se in order to experience good health. These results indicate that in order to promote good health it might be important to ask questions both about positive and negative issues. For example, the visitor could support the person by having a dialogue about how the person could maintain energy and endurance. Having a dialogue about the absence of a problem might lead to insights about the person’s own assets and abilities to maintain or improve health. Thus, those who conduct the PHVs need to listen to the answers concerning whether there is a problem or not, and with that information promote health. These results demonstrate the importance of using a comprehensive approach including both health promotion and risk prevention in order to maintain or improve health [6]. However, as Bauer et al. [6] indicate, in order to support older person’s ability to manage and experience good health, mental and physical factors on a societal level also need to be considered.

With the results from the present study it is possible to gain a deeper understanding of how society can support healthy ageing with public health interventions targeting factors associated with good health among older persons. The present results showed that mental and physical factors are important for the person’s ability to feel valuable and participate in social activities. Similar results have been found in other studies. A systematic literature review showed that social activities have a positive impact on older persons’ health [4], and feeling a purpose in life has been identified as an asset that can contribute to good health [8]. Further, mental and physical factors were recognised as important for health as early as the 1940s when they were part of the WHO’s definition of good health [44]. However, there are other definitions of health, for example that of Huber et al. [12] where health is determined by the ability to adapt and manage challenges; thus it also includes persons that experience good health despite coping with illnesses. Moreover, in order to meet the demands of the SDG 2030 which highlights the need for a health promotion perspective that includes all, society needs to adapt a health promotion perspective that also includes older persons [10, 11]. By applying an asset-based approach [7, 9] the risk-focused questions asked during PHVs can be turned into health assets and taken into account on a societal level, which can guide the development of an age-friendly society. With the knowledge from the present study, society could consider how to create an environment that helps older persons to do things that make them feel valuable and enables them to participate in social activities. There is a need for public health intervention focusing on health promotion targeting older persons such as PHVs. This could then enable society to empower older persons to strengthen and develop their assets and capacity to cope with challenges and experience good health.

This was a register study with a cross-sectional design that imposed some challenges. Obtaining items from a register means that the items considered in the register were not defined by the researcher and not purposely made for research. In order to facilitate the dialogue during the PHV and minimise the number of questions asked by the district nurse, single items were used. Previous research has shown that single items could make answering questions easier for the respondent, because fewer questions have to be asked and at the same time be valid and reliable for different health issues [35, 45]. The single-item SRH is a good example of a usable single item [35, 36]. Another limitation is that the items used were not validated measures, which could give misleading results. The items were dichotomised, which can increase the risk of missing nuances in the result; however, this was done due to low frequencies of responses in some response alternatives, and also to facilitate the interpretation of the data. Furthermore, in a cross-sectional study like this nothing could be said about the causal relationship between the items. A strength of the present study was that experienced district nurses were conducting the PHVs using a well-established model. However, information bias could have occurred since nurses might have observed and recorded information differently. Strengths of the present study were that the sample was large, and the number of internal missing observations was rather low. The results are generalisable to older persons with no or minimal home care, aged 75 years or older. A limitation was that information about non-participants was lacking.

## Conclusions

The main conclusion of this study is that questions focusing on risks could be seen from a health promotion perspective and thereby be turned into assets having a positive impact on older persons’ health. Furthermore, both the mental and physical factors identified in the results as associated with good health have implications for the person’s ability to feel valuable and participate in social activities. A comprehensive approach is needed to tackle the challenges that come with ageing and to help older persons to strive for good health. Positive factors as well as factors indicating the absence of problems could be considered on an individual level as well as on a societal level to improve or maintain health among older persons. The results suggest that issues regarding both health promotion as well as risk prevention must be brought up during the preventive home visit.

## Supplementary information

**Additional file 1.** Description of the items.

## Data Availability

The data that support the results were used under the ethical permission for the current study and are not publicly available due to confidentiality. However, data are available from the authors upon reasonable request.

## Abbreviations

ADL: Activities of Daily Living
CI: Confidence interval
PHV: Preventive home visit
POR: Positive odds ratio
SDG: Sustainable development goals
SRH: Self-rated health
WHO: World Health Organization
