# Supplementary material for: Factors associated with good health among older persons who received a preventive home visit: a cross-sectional study
Source: BMC Public Health. 2020 May 14;20:688. doi: 10.1186/s12889-020-08775-6 (PMC7227102; doi:10.1186/s12889-020-08775-6)
Supplement: Supplementary file 1 — Additional file 1. Description of the items. [file 12889_2020_8775_MOESM1_ESM.pdf]

Additional file 1 Description of the items.

Items	Question	Answer	Dichotomised
**Self-rated health (SRH)**	In general, how would you describe your health and wellbeing?	Excellent, Very good, Good, Fair or Bad.	**Good** (the first three alternatives) =1<br>**Poor** (the last two alternatives)=0
**Demographics**			
**Age**	What year were you born?	Year	
**Gender**	Male or Female	**Male**=0<br>**Female**=1	
**Settlement**	Rural or Urban. (Definition of ‘urban’ is a town with more than 200 inhabitants)	**Urban**=0<br>**Rural**=1	
**Cohabitation**		Cohabitant, Alone or Living apart	**Yes** (the first alternative) =1<br>**No** (the two last alternatives) =0
**Satisfied with accommodation**	Are you satisfied with your accommodation?	**Yes**=1<br>**No**=0	
**Safe neighbourhood**	Do you feel safe in your neighbourhood?	**Yes**=1<br>**No**=0	
**Enough money**	Do you have enough money to pay your bills?	**Yes**=1<br>**No**=0	

**Worried about finances**	Are you worried about your finances?	**No**=1<br>**Yes**=0	
**Mental factors**			
**Safe/serenity/harmony**	How much of the time do you feel safe/serenity/harmony?	All the time, Sometimes or Never	**All the time** (The first alternative)= 1<br>**Sometimes** (the two last alternatives)=0
**Good cognition**	In general do you think that your memory is good in comparison to others of your age?	**Yes**= 1<br>**No**=0	
**Sleep**	Do you experience good sleep?	**Yes**=1<br>**No**=0	
**Loneliness**	Are you bothered about loneliness?	No, Sometimes or Yes	**No** (the first alternative) =1<br>**Yes** (the two last alternatives)=0
**Sad/gloomy**	How much of the time do you feel sad/gloomy?	Never, Sometimes or All the time	**Never** (the first alternative)=1<br>**Sometimes** (the two last alternatives)=0
**Anxiety/worried**	How much of the time do you feel worried/anxiety?	Never, Sometimes or All the time	**Never**(the first alternative)=1<br>**Sometimes** (The two last alternatives)=0

**Future**	When I think about the future I feel…	…not at all worried,<br>…little worried,<br>…very worried.	**Not worried** (the first alternative) =1<br>**Worried** (the two last alternatives) =0
**Influence on own situation**	Do you feel that you have the influence you wish over your own situation?	Yes, Partly or No	**Yes** (the first alternative)=1<br>**No** (the two last alternatives) =2
**Influence on society**	Do you feel that you have the influence you wish over society?	Yes, Partly or No	**Yes** (the first alternative)=1<br>**No** (the two last alternatives) =2
**Satisfied with life**	Do you feel satisfied with your life in general?	Yes, Fair or No	**Yes** (the first alternative) =1<br>**No** (the last two alternatives)=2
**Ability to do things that make you feel valuable**	Do you feel that you can do things that make you feel valuable?	I can do everything that makes me feel valuable,<br>I can do a lot that makes me feel valuable,<br>I can do little that makes me feel valuable, or	**Yes** (the two first alternatives)=1<br>**No** (the two last alternatives) =0

		I cannot do anything that makes me feel valuable	
**Tiredness/reduced energy**	Have you experienced tiredness/reduced energy in the last three months?	**No**=1<br>**Yes**=0	
**Physical factors**			
**Impaired endurance**	Have you experienced impaired endurance? (Do you get tired after a short walk of 15 minutes?)	**No**=1<br>**Yes**=0	
**Good Activities of Daily Living (ADL)**	Do you need help with shopping?	Yes, Partly or No	**No** (the last alternative)=1<br>**Yes** (the two first alternatives) = 0
**Physical problems hindering social participation**	Do you have any physical problem that is hindering you in social contexts?	**No**=1<br>**Yes**=0	
**Pain**	Are you bothered about pain?	**No**=1<br>**Yes**=0	
**Urinary continence**	Are you bothered about urinary incontinence?	**No**=1<br>**Yes**=0	
**Digestive problems**	Do you have problems with your tummy?	**No**=1<br>**Yes**=0	

**Vision**	Do you experience problems with you vision?	**No**=1<br>**Yes**=0	
**Hearing**	Do you experience problems with your hearing?	**No**=1<br>**Yes**=0	
**Lifestyle factors**			
**Physical activity**	Do you do any physical activity, for example walking, cycling, housekeeping or gardening?	Daily, Several times a week, Once a week, One to three times a month or Never/ almost never	**Several times per week** (two first alternatives) =1<br>**Rarely** (the three last alternatives)=0
**Appetite**	How is your appetite compared to before?	Better, The same or Worse	**Good** (the two first alternatives)=1<br>**Poor** (the last alternative) =0
**Weight loss**	Have you lost weight (unintentional)?	**No**=1<br>**Yes**=0	
**Alcohol**	Do you drink alcohol?	Never, Rarely, Sometimes or Frequently	**Rarely** (the two first alternatives)=1<br>**Frequently** (the two last alternatives) =0
**Smoking/snuff**	Do you smoke or use snuff?	No, snuff, Smoke or Both snuff and smoke.	**No** (the first alternative)=1<br>**Yes** (the last three alternatives)=0

**Use of smartphone**	Do you use a smartphone?	**Yes**=1<br>**No**=0	
**Use of computer**	Do you use a computer?	**Yes**=1<br>**No**=0	

Permission received from the project leader (Pia Petersson, Ph.D.) for ‘Preventive home visits to seniors’ to publish this part of the questionnaire in full version.
